# Commentary: The longitudinal impact of emotional intelligence and psychological empowerment on work engagement among university administrators: a cross-lagged panel model approach

**DOI:** 10.3389/fpsyg.2026.1834728

**Published:** 2026-07-08

**Authors:** Kimmo Sorjonen, Bo Melin, Marika Melin

**Affiliations:** Karolinska Institutet, Department of Clinical Neuroscience, Stockholm, Sweden

**Keywords:** emotional intelligence, psychological empowerment, spurious prospective effects, statistical scrutiny, work engagement

Zhou and Wang (2026) analyzed data on self-rated emotional intelligence, psychological empowerment, and work engagement, collected from university administrators in China (*N* = 416, mean age = 31.6 years, 52.5% women) on two occasions 6 months apart. Based on statistically significant cross-lagged effects (e.g., of initial emotional intelligence on subsequent work engagement when adjusting for initial work engagement), Zhou and Wang concluded that both emotional intelligence and psychological empowerment contribute to increased engagement among university administrators.

## Cross-lagged effects may be spurious

**Figure 1:**
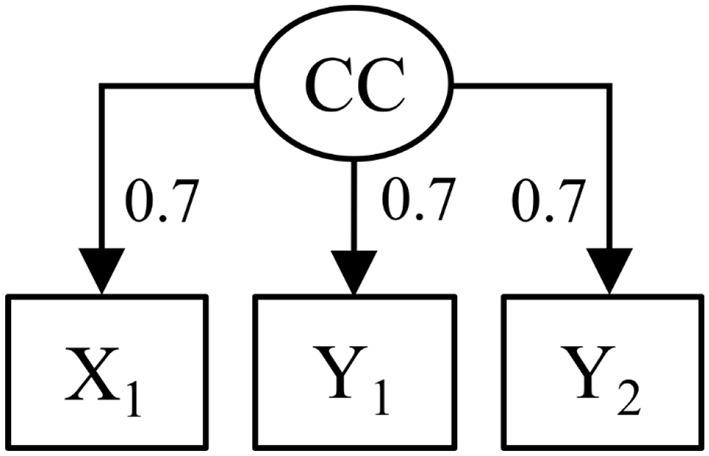
Model that would generate data with a spurious effect of X_1_ on Y_2_ when adjusting for Y_1_ (β = 0.33). CC, common cause.

However, it is well-known that adjusted cross-lagged effects may be spurious (e.g., Campbell and Kenny, 1999; Sorjonen et al., 2019). For example, if data is generated as in Figure 1, the effect of X_1_ on Y_2_ when adjusting for Y_1_ would be β = 0.33 although there is no direct effect of X_1_ on Y_2_, meaning that the effect would be spurious.

We (Sorjonen et al., 2025b) have recommended researchers to scrutinize cross-lagged effects by estimating the effect of X_1_ on the Y_2_-Y_1_ difference score when adjusting for Y_1_ (coefficient *a*_1_ in Equation 1), when adjusting for Y_2_ (coefficient *b*_1_ in Equation 2), and when not adjusting for Y_1_ or Y_2_ (coefficient *c*_1_ in Equation 3). Then, these three effects may be meta-analytically pooled and conclusions based on the pooled effect. It should be noted that all three effects are of X_1_ on subsequent change in Y. Our proposed procedure is in line with, and inspired by, multiverse methodology, where effects of interest are estimated in several plausible models and conclusions based on a juxtaposition of the findings (Steegen et al., 2016). Moreover, it has been argued that it is feasible to aggregate multiverse findings meta-analytically (Bartoš et al., 2025). Using this, or similar, methodology we have shown, for example, that prospective cross-lagged effects of various predictors on burnout and exhaustion (Sorjonen et al., 2025a), of self-esteem on anxiety and academic self-efficacy (Sorjonen and Melin, 2025b), and between grip strength and cognitive function (Sorjonen and Melin, 2025a; Sorjonen et al., 2026b), reported in the research literature, may have been spurious. Here, we used this methodology to scrutinize the findings and conclusions by Zhou and Wang.


$$\begin{array}{lll} Y_{2}-Y_{1} & = & a_{0}+a_{1}X_{1}+a_{2}Y_{1}+e \end{array}$$ (1)

$$\begin{array}{lll} Y_{2}-Y_{1} & = & b_{0}+b_{1}X_{1}+b_{2}Y_{2}+e \end{array}$$ (2)

$$\begin{array}{lll} Y_{2}-Y_{1} & = & c_{0}+c_{1}X_{1}+e \end{array}$$ (3)

## Our reanalyses

We estimated three effects (*a*_1_, *b*_1_, and *c*_1_ in Equations 1–3, respectively) in data simulated to resemble the data used by Zhou and Wang, with the same sample size and correlations between variables. Simulations were carried out with the mvrnorm-function in the MASS package with correlations reported by Zhou and Wang (see their Table 3) as input. Exact details of the simulation, as well as the correlation matrix, are available in our analytic script (see link below). Then, these three effects were pooled by random effects meta-analytic models. Analyses were conducted separately for the effect of emotional intelligence on work engagement and for the effect of psychological empowerment on work engagement, respectively. We used simulated data as the empirical data was not available to us. The corresponding author of the study by Zhou and Wang did not respond to our request for the data. Here, it is important to note that standardized regression effects are functions of correlations. Given standardized (*M* = 0, *SD* = 1) variables X_1_, Y_1_, and Y_2_, effects *a*_1_, *b*_1_, and *c*_1_ in Equations 1–3 are given by Equations 4–6 (which are based on standard equations for adjusted regression effects, see Cohen et al., 2003), respectively. This means that the effects are fully defined by correlations between the variables (*r*_*x*1, *y*1_*, r*_*x*1, *y*2_, and *r*_*y*1, *y*2_, respectively) irrespective of other complexities in data, e.g., distributions of the variables, measurement error, and potential respondent heterogeneity. Hence, the findings reported below approximates what the findings would have been if estimated in the empirical data used by Zhou and Wang. Data simulations and analyses were conducted with R 4.5.3 statistical software (R Core Team, 2026) employing the MASS (Venables and Ripley, 2002), metafor (Viechtbauer, 2010), and lavaan (Rosseel, 2012) packages. The analytic script, which also generates the used data, is available at the Open Science Framework at https://osf.io/68ras/.


$$\begin{array}{lll} a_{1} & = & \frac{r_{x1y2}-r_{x1y1}r_{y1y2}}{1-r_{x1y1}^{2}} \end{array}$$ (4)

$$\begin{array}{lll} b_{1} & = & \frac{r_{x1y2}r_{y1y2}-r_{x1y1}}{1-r_{x1y2}^{2}} \end{array}$$ (5)

$$\begin{array}{lll} c_{1} & = & r_{x1y2}-r_{x1y1} \end{array}$$ (6)

**Figure 2:**
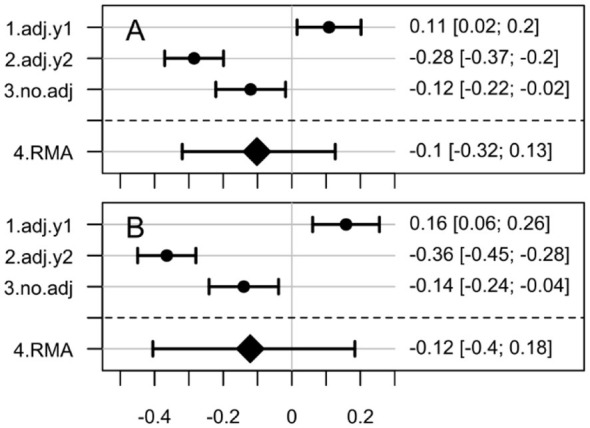
**(A)** Effects [with 95% CI] of initial emotional intelligence on subsequent change in work engagement; **(B)** Effects of initial psychological empowerment on subsequent change in work engagement. Effects with number 1 are adjusted for an initial score on work engagement, effects with number 2 for a subsequent score on work engagement, and effects with number 3 neither for an initial nor a subsequent score on work engagement. Effects 1–3 correspond to coefficients *a*_1_, *b*_1_, and *c*_1_ in Equations 1–3, respectively. Effects with number 4 are random model meta-analytic (RMA) aggregations of effects 1–3.

In line with results reported by Zhou and Wang, initial emotional intelligence and psychological empowerment had positive (i.e., increasing) effects on subsequent change in work engagement when adjusting for initial work engagement (effects with number 1 in Figures 2A, B, respectively, corresponding to coefficient *a*_1_ in Equation 1). However, if adjusting for subsequent instead of initial work engagement, or for neither initial nor subsequent work engagement, these effects were, paradoxically, decreasing (effects with number 2 and 3 in Figures 2A, B, corresponding to coefficients *b*_1_ and *c*_1_in Equations 2 and 3, respectively). Meta-analytic poolings of these discrepant effects were negative but not statistically significant (effects with number 4 in Figures 2A, B, respectively).

## The spurious prospective associations model (SPAM)

**Figure 3:**
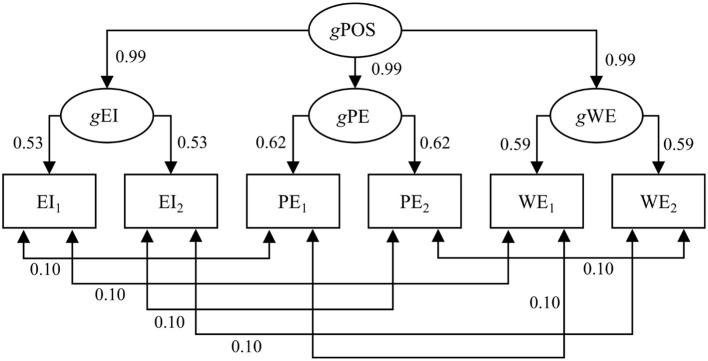
The spurious prospective associations model (SPAM), where scores on emotional intelligence (EI), psychological empowerment (PE), and work engagement (WE) on two occasions (the subscripts) are affected by general levels of the constructs (*g*EI, *g*PE, and *g*WE, respectively) which are, in turn, affected by general positivity (*g*POS). Correlated residuals account for impact by common state factors. Standardized estimates. Parameters with the same value were constrained to equality. The model had excellent fit [χ^2^ = 20.3, *DF* = 17, *p* = 0.258, CFI = 0.994, TLI = 0.995, RMSEA = 0.022 [90% CI: 0.000; 0.052]].

Instead of assuming that emotional intelligence and psychological empowerment had genuine (i.e., non-spurious) increasing prospective effects on work engagement, a conclusion that would be contradicted by the findings reported above, we propose that data analyzed by Zhou and Wang, and reanalyzed by us, may have been generated by the spurious prospective associations model (SPAM, Sorjonen et al., 2025c) (Figure 3). In the SPAM, longitudinal scores on constructs are affected by general levels of the constructs and these are, in turn, affected by some overarching trait factor. Here, this trait factor could be, for example, general positivity. Correlated residuals between scores measured at the same timepoint account for a potential impact by some common state factor, e.g., temporary mood. However, the SPAM does not include any direct effects between the longitudinal scores.

The SPAM had excellent fit [χ^2^ = 20.3, *DF* = 17, *p* = 0.258, CFI = 0.994, TLI = 0.995, RMSEA = 0.022 [90% CI: 0.000; 0.052]]. This suggests that the data analyzed by Zhou and Wang may have been generated without direct effects between scores on emotional intelligence, psychological empowerment, and work engagement and that the cross-lagged effects reported by Zhou and Wang, therefore, may have been spurious and their conclusions incomplete.

## Limitations

It should be noted that the present findings do not prove, once and for all, that emotional intelligence and psychological empowerment do not have any genuine (i.e., non-spurious) effects on work engagement. The more limited conclusion to be drawn is that the data used by Zhou and Wang, and simulated and reanalyzed by us, does not prove that the null hypothesis of no genuine effects is false. Similarly, the good fit does not prove that the SPAM was the true data generating model, as other models may also fit the data well. Rather, the good fit indicates that the SPAM may have generated the data.

The instruments used to measure emotional intelligence, psychological empowerment, and work engagement may not have been optimal. However, it should be kept in mind that such factors were constant across the analyzed models, as they were fitted to the same data, and could, therefore, not explain why the models indicated discrepant increasing and decreasing effects. We do not believe that it would be tenable to argue that due to potential methodological shortcomings we should trust the findings and conclusions of genuine increasing effects by Zhou and Wang and ignore the contradicting effects, and null meta-analytic effects, reported here. On the contrary, potential methodological shortcomings would speak, in our opinion, for rather than against our recommendation to postpone strong claims and conclusions.

The random-intercept cross-lagged panel model (RI-CLPM) is an extension of the traditional cross-lagged panel model (CLPM), where autoregressive and cross-lagged effects are estimated while adjusting for individuals' general trait-like levels of the constructs (Hamaker et al., 2015; Mulder and Hamaker, 2021). Hence, effects are estimated within individuals rather than between individuals as in the traditional model. Within-individual effects are often assumed to be better indicators of potential causality (Hamaker et al., 2015; Usami et al., 2019). However, the RI-CLPM could not be used here, as it requires data from at least three waves of measurement while the present data only included two waves. Moreover, the RI-CLPM does not adjust for time-varying confounders (Rohrer and Murayama, 2023; Murayama and Gfrörer, 2024). This means that the RI-CLPM is susceptible to similar spurious findings as the traditional CLPM and may be contradicted by findings by other models (Sorjonen and Melin, 2025c; Sorjonen et al., 2026a,c). Hence, even if it could have been used in the present case, the RI-CLPM would not have been able to provide irrefutable evidence of causality.

## Summary and concluding remarks

Here, we scrutinized findings and conclusions by Zhou and Wang, suggesting that emotional intelligence and psychological empowerment contribute to increased work engagement among university administrators. In reanalyses of data generated to resemble the data used by Zhou and Wang, we found discrepant increasing and decreasing prospective effects depending on the analyzed model. Meta-analytic poolings of these discrepant effects did not differ significantly from zero. Hence, the findings by Zhou and Wang may have been spurious and their conclusions incomplete. A conclusion of spuriousness would be supported by the good fit of the SPAM, which does not assume direct effects between longitudinal scores on constructs.

It is important for researchers to bear in mind that correlations, including adjusted cross-lagged effects, in observational (i.e., non-experimental) data may be spurious rather than due to genuine effects. Otherwise, researchers risk overinterpreting findings, something that may have happened to Zhou and Wang. We recommend researchers, e.g., in organizational psychology, to scrutinize cross-lagged effects by fitting, as we did here, alternative models to data and to base conclusions on a juxtaposition of findings. Our analytic script, available at https://osf.io/68ras/, may be modified and used with other datasets. If findings in alternative models align, conclusions can be drawn with increased confidence. However, if effects diverge, like in the present study, confident conclusions and policy recommendations should be postponed.
